# The Impact of Working Environment on Nurses' Caring Behavior in Sabah, Malaysia

**DOI:** 10.3389/fpubh.2022.858144

**Published:** 2022-04-07

**Authors:** Norkiah Arsat, Bee Seok Chua, Walton Wider, Norsimah Dasan

**Affiliations:** ^1^Faculty of Medicine and Health Sciences, Universiti Malaysia Sabah, Kota Kinabalu, Malaysia; ^2^Faculty of Psychology and Education, Universiti Malaysia Sabah, Kota Kinabalu, Malaysia; ^3^Faculty of Business and Communication, INTI International University, Nilai, Malaysia

**Keywords:** work environment, caring behavior, public hospitals, nurse, Sabah

## Abstract

**Aims:**

This study aims to investigate 5 types of work environment influencing nurses' caring behavior, namely (i) participation in hospital affairs, (ii) foundations for quality of care, (iii) manager ability, leadership, and support of nurses, (iv) staffing and resource adequacy, and (v) nurse-physician relations.

**Design:**

This research is a cross-sectional study using the survey method.

**Methods:**

Data were collected from 3,532 nurses working in public hospitals and health clinics within Sabah, Malaysia in 2015. The hypothesized model was evaluated using partial least squares method.

**Results:**

The findings reveal that all forms of work environment have a positive effect on nurses' caring behavior except for staffing and resource adequacy which shows a negative effect on caring behavior.

**Conclusion:**

Overall, this study has added to theoretical contributions in the academic and research fields as well as in practical implications in the field of nursing practice by addressing the influence of work environments on caring behavior.

**Implications for Nursing Management:**

The present research has provided convergent evidence on the role of the working environment in influencing the behavior of nurses working in hospitals and health clinics in Sabah, Malaysia.

## Introduction

The concept of caring services was first introduced by Malaysia Ministry of Health (MOH) in 1987 through its corporate culture. Since then, caring behavior has become a core value that needed to be put into practice by all staff in the public sector health services (1). Caring service practices include having a friendly attitude, being attentive, providing service courteously and responsively, and being respectful of individual rights. The application of these values equates to realizing the vision and mission of MOH, which is to promote and facilitate the use of health services in the community in order to achieve optimal health and a high quality health system (2). To achieve this effort, MOH organized training for staff toward developing a caring culture, professionalism and teamwork. This endeavor has to some extent seen a change in the attitude and behavior of Ministry of Health employees since several years ago; they have become more courteous, responsive, respectful, and friendly to customers. Nurses make up the largest workforce in the public hospital and public health services, and they spend 24 h with patients and clients, making them more significant than other health personnel in terms of satisfaction toward staff caring behavior (3, 4). Although various ways and efforts to improve caring services have been implemented, complaints of dissatisfaction with the healthcare services provided still remain (5). In fact, the Health Ministry receives an average of 7,000 complaints annually, covering various health aspects, such as services and facilities (6). There are complaints of unfriendly nurses going about their work indifferently, and even berating or sneering at women in painful labor (7). It is crucial, therefore, to identify the factors affecting caring behavior among MOH nurses that may affect the client's satisfaction with the healthcare services provided by MOH.

## Literature Review

### Nurses' Caring Behavior

Caring behavior makes up the philosophical and ethical foundation for professional nursing, and is a major focal point in nursing which is regarded both an art and a science. This underpinning offers a framework that takes up and cuts across art, science, humanities, spirituality, and new dimensions of mind-body-spirit medicine. Nursing has openly evolved as central to the human phenomenon of nursing practice (8). Nurses' professional nursing practice is implemented through direct and indirect nursing care (9). Direct nursing care is the most prioritized in nursing practice and can be observed from nurses' behavior, quality of nursing care and patients and clients' outcomes. Nurse-patient caring includes dimensions such as respectful deference to others, assurance of human presence, positive connectedness, professional knowledge and skills, attentiveness to the other's experience (10). But the fact is that nurses have limited time to interact with patients as they are mostly involved in routine tasks, such as pushing the doctor's trolley around, preparing patients' files for doctors and specialists' visits, retrieving, reviewing and implementing doctors and specialists' instructions, etc. (11). Thus, the chance to implement direct nursing care and apply caring behavior is limited, as the time available has been diverted to indirect patient care activities. A meta synthesis of nurse caring by Finfgeld-Connett (12), indicated that a conducive work environment has been found to influence caring behaviors. Work environment is described as the organizational characteristics of a work setting that eases or hampers professional nursing practice (13). According to Hughes (14), “the work environment in which nurses provide care to patients can determine the quality and safety of patient care. As the largest healthcare workforce, nurses apply their knowledge, skills, and experience to care for the various and changing needs of patients. A large part of the demands of patient care is centered on the work of nurses.” This fundamental understanding to professional nursing practice has rarely been given attention with regard to its role in ensuring quality patients' outcomes. The researcher intends to highlight nurses' caring behavior that has been identified as the foundation of professional nursing practice, and examine its relationship with the work environment. Factors that influence nurses' caring behavior in nursing practice deserve study because nurses' behavior determines their performance and patients' outcomes. Poor performance will affect quality of patient care which in turn affects the clients' satisfaction with the care they have received from nurses.

### Underpinning Model

It can be argued that there are underlying barriers for nurses to practice caring behavior in the healthcare organization (15). Watson (16) stated that the focus of caring and economic models contradict caring and administrative practices. Dominant economic models generally focus on bed and disease, physiology as entity, technology, and products as short-term solutions to patient care needs. To overcome the shortage of nurses, they focus on incentives, such as increasing enrollments, giving bonuses, offering relocation fees, etc., and not on addressing underlying dissatisfaction, for example the inability to professionally perform direct-care, person centered, human-to-human relationships and caring-healing processes and practices. This void in caring persists in spite of corporate rhetoric and slogans of “caring institutions.” The pressure that comes with this tends to divert nurses' behavior from its original intention, and prevent them from practicing the behavior and tasks that had drawn them to this noble profession initially. The result is a nurse working in a work environment that is not conducive and dominated by economic concerns that emphasize profit rather than quality of nursing care and healing experiences. In that regard, poor working conditions unbefitting complex nursing care provision, may indicate a lack of caring (17).

**Figure 1:**
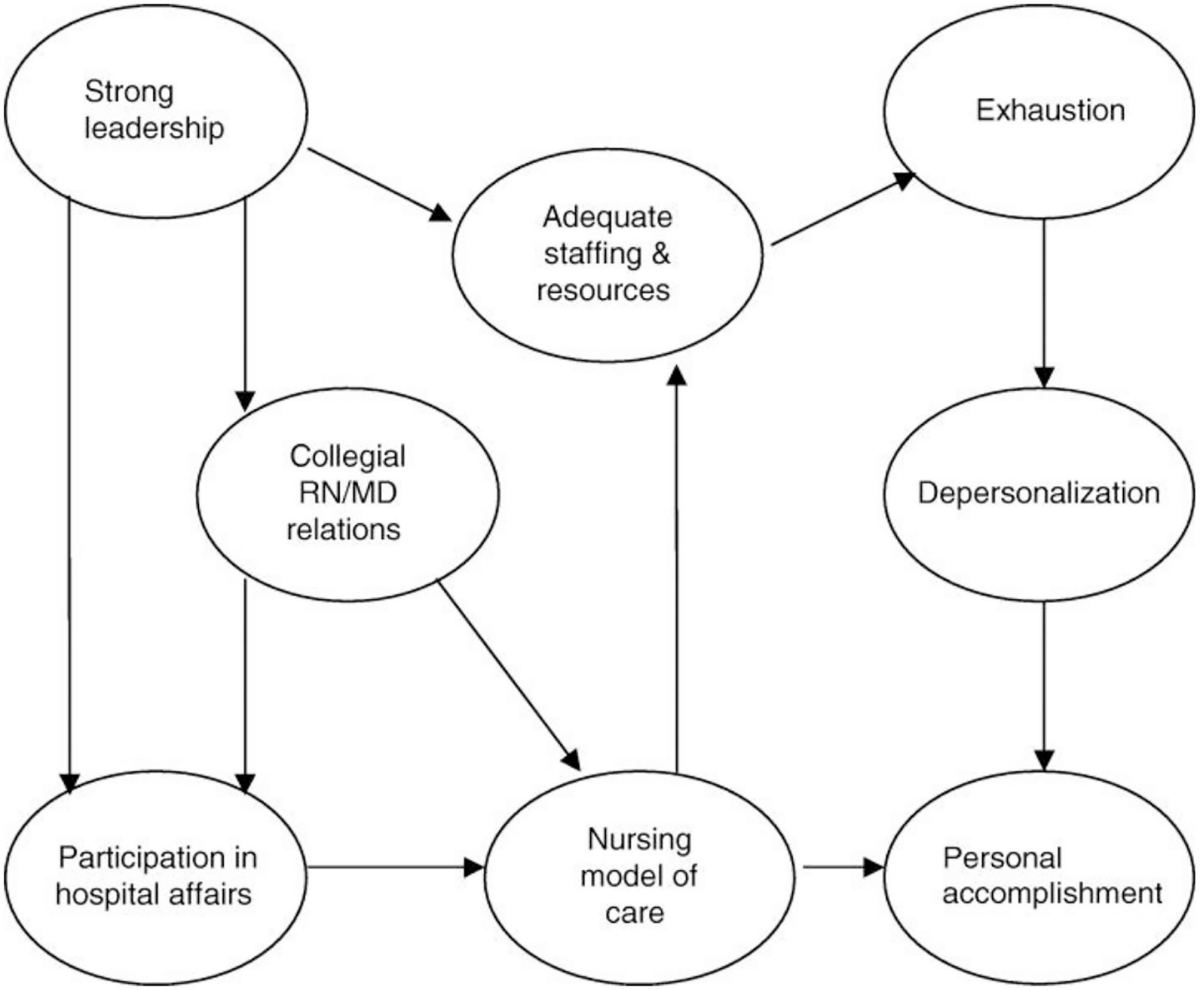
The Nursing Work-Life Model. Source: Manojlovich and Laschinger (21).

Roche et al. (18) suggested that the Nursing Work-Life Model (NWLM) can be applied in the nursing work environment globally in terms of factors that are required in the work environment to enable nurses to provide quality patient care. The NWLM was developed to explain how an organizational or nursing unit influences and affects nurses' lives in the workplace by either contributing to or mitigating burnout (19). The NWLM identified five characteristics of nurses' working culture in a professional nursing practice environment that effectively interact with one another and affect the outcomes through the burnout/engagement process (20, 21). The first two subscales, Nursing Participation in Hospital Affairs and Nursing Foundations for Quality of Care, appear to reflect the hospital-wide environment. The latter three subscales, Nursing Manager Ability, Leadership, and Support, Staffing and Resource Adequacy, and Nurse–Physician Relations, are more likely to be unit specific (13). Figure 1 illustrates the Original NWLM.

### Work Environment and Caring Behavior

The literature has identified that the most commonly used instrument to measure the work environment is the Practice Environment Scale of the Nursing Work Index (PES-NWI) developed by Lake (13) Zangaro and Jones (22). The nursing practice environment scale (PES) represents factors that allow nurses to practice to the full scope of clinical practice and deliver safe, quality care to patients (13). The PES-NWI has been endorsed as a favorable measure of the nursing practice environment in Malaysian public hospitals (3). Positive working environments are typified by cohesive teams, collegial relationships, adequate staffing, professional development, and supportive leadership (23). Where health clinics are concerned, however, there are staffing and resource adequacy problems (24), and nurses are unable to spend enough time with clients as there are insufficient nurses to provide quality healthcare. Nurses' workload has increased due to the expanded and extended scope of the healthcare program, data collection, and paperwork to be done like keeping the necessary records and reports. Other than that, nurses are often used as assistants to other health professionals, doing work like informing clients of their turn to be examined by doctors and medical assistants, and documentation for the clients' attendance in outpatient units. This results in an increased workload for nurses, and subsequently, they have limited time to implement proper healthcare and health education for their clients. This extra workload may cause nurses to focus less on essential tasks, conflict among nurses and other healthcare staff, and emotionally disturbed and negative behavior that affects nurse-client relationships, ultimately contributing to poor delivery of nursing care. All this will result in patients and clients feeling dissatisfied with the nurses, prompting them to lodge complaints.

Although abundant studies have been conducted to investigate the relationships between the domains of the PES-NWI with CBI-24 (25), the results are inconsistent (26). One such example is a study conducted by Persky et al. (27) where they reported that poorer work environments were associated with higher levels of caring. The findings appear incongruent with the Aiken et al. (28) study which reported that better patient care environments were strongly associated with nurses' perceptions of quality of care.

Laschinger and Leiter (29) found that nurses' leadership play a vital role in the quality of their work-life, with regard to involvement on policy, staffing, support for nursing care (vs. medical), and the nurse/physician relationship. Ahn et al. (30) reported that the nursing foundations for quality of care were significant predictors of caring behavior among Korean clinical nurses. Past studies also found that the nurses' perception of leadership in their units was significantly and directly associated with their perceived participation in decision-making, staffing and resources, and the quality of nurse-physician relationships (18, 31). The relationship between leadership and decisional involvement was found to be very strong, and in turn strongly influenced the perceived use of a nursing-based (vs. medical) model of patient care, and perceptions of resource adequacy to support quality patient care. The findings have highlighted the role of leadership as important in supporting nurses' work environment. In addition, several studies have reported that when nurses perceive their work environment as positive and supportive, such that the work environment constitutes crucial essentials such as adequate staffing, allowing nurses to participate in hospital affairs, nurse manager support, encouraging good nursing service, and formulating good nurse–physician relationships, they would experience an obligation and strong urge to reciprocate favors received from the organization by giving quality care services to the patients (25, 32–36).

Several studies done on the caring behavior of public healthcare workers from 1995 to 2006 in Malaysia (2) involved medical assistants, medical officers, and staff as a whole. However, studies on caring behavior specifically of nurses in Malaysia are limited (4). Generally, nurses are required to carry out tasks efficiently to ensure that clients receive quality healthcare services, thus giving satisfaction and better outcomes to both clients and healthcare organizations. Yet, not much attention has been given to how well the work environment where nurses perform daily nursing activities support the latter to best meet job demands and maintain good performance for quality care outcomes (3). Specifically, this study intends to fill the gap by examining the effects of the working environment, in relation to participation in hospital affairs; foundations for quality of care; manager ability, leadership, and support of nurses; staffing and resource adequacy; and nurse-physician relations, on nurses' caring behavior in Sabah public hospitals and public health services.

This study aims to investigate 5 types of work environment influencing nurses' caring behavior. Hence, we hypothesized that:

Hypothesis 1: Nursing participation in hospital affairs has a positive direct effect on caring behavior.
Hypothesis 2: Nursing foundations for quality of care have a positive direct effect on caring behavior.
Hypothesis 3: Nursing manager ability, leadership, and support of nurses of nurses have a positive direct effect on caring behavior.
Hypothesis 4: Staffing and resource adequacy has a positive direct effect on caring behavior.
Hypothesis 5: Nurse-physician relations has a positive direct effect on caring behavior.

## Methods

### Design

This research is a cross-sectional study using the survey method to examine the relationship between nurses' working environment and nurses' caring behavior.

### Participants

There are 10,637 registered nurses in Sabah state, encompassing all categories of nurses (37). The location of the study is comprised of public hospitals and public health services throughout Sabah. The respondents of the study were registered nurses of all categories in Grades, as well as the management and professional group, who served in public hospitals, and public health services (health clinics, maternal and child health clinics, rural clinics, traveling clinics, and 1 Malaysia Clinics) throughout the state of Sabah.

### Ethical Considerations

Ethical consideration and approval to conduct the study in hospitals and district health offices had been obtained from the Medical Research and Ethics Committee (MREC), and National Medical Research Register (NMRR) (ref. no: NMRR-14-1226-21410) of Malaysia, Ministry of Health (KKM) and Sabah State Health Director [ref no: JKN(SB)PJNS/32].

### Data Collection

This study used multistage cluster sampling to collect data. At the first stage, multistage cluster sampling was used to choose hospitals and district health offices. Among these hospitals and district health offices, a random cluster sampling was used to select the larger hospitals that had many wards and units, and district health offices that had many health clinics, rural clinics, and other units in order to collect sufficient data from nurses for the total suggested sample size. The researchers chose seven district health offices out of 24 district health offices in the state. Within each district, health clinics were chosen using random cluster sampling involving 10 health clinics, nine maternal and child health clinics, 73 rural clinics, and three traveling or mobile clinics. As for hospitals, the researchers selected a total of 12 hospitals with a total of 244 wards and units out of 24 hospitals across the state.

At the second stage, the sample was clustered according to wards or units in hospitals and health clinics, rural clinics, and other units in the public health services. Subsequently, a simple random sampling was used to select the wards and units with a larger number of nurses, and for health clinics, rural clinics and units in the public health services, a simple random sampling was used to select those that had a larger number of nurses for distribution of questionnaires.

Prior to data collection, the researchers met with every hospital director, hospital matrons, area health officers, and district health matrons to discuss the administering of questionnaires. They proposed that the questionnaires be administered by the nursing sister or nurse-in-charge to avoid disruption to the nurses on duty. The questionnaires were distributed through the Nursing Administration Unit [Matrons, and Head Nurse (Nursing Sister)] in public hospitals (wards and units), and public health services (health clinics, maternal and child health clinics, rural clinics, traveling clinics, and 1 Malaysia Clinics) throughout the state. All personnel involved in the data collection procedure were briefed on how to explain the purpose, confidentiality of the study, how to collect the data, and how to respond to any respondents' inquiries. The personnel involved in the data collection procedure were also required to inform the respondents that they had the right to decline answering any question for any particular reason, or withdraw from the study at any time. Completed questionnaires were kept in sealed envelopes or sealed paper boxes to ensure confidentiality and were not accessible to anyone.

To collect completed questionnaires, the researchers and research assistants re-visited each research site, though some officers, matrons, nursing sisters, and nurses were kind enough to volunteer to send the completed questionnaires by mail or through officially recognized individuals. Nevertheless, some challenges arose in the collection process. First, the geographical location of hospitals throughout the state is such that road access is difficult and takes time, especially for health clinics which are mostly located in remote areas. Also, several persons tasked with the responsibility for making decisions to collaborate in certain hospitals and health clinics and allow data collection to be implemented were unable to do so, although researchers had met with them previously and explained the purpose of the study together with evidence of ethical considerations obtained from the MOH. Given this, the researchers had to change the location of the study to the nearest hospital or clinic willing to participate.

Notwithstanding that, a total of 4,000 questionnaires were distributed to the respondents from May to October, 2015. The response rate was *n* = 3,867 (96.68%). However, during the process of data entering, two questionnaires were found not filled, three questionnaires were unusable due to missing data, and three questionnaires had similar responses presumably filled by the same respondent. Next, straight lining was identified in 327 responses. According to Hair et al. (38), straight lining happens when a respondent gives a high rate of same responses in the questionnaire, causing a bias response to the data. This brought the total number of questionnaires that could be used to *n* = 3,532. That means the actual response rate was 88.3% which was considered a very high response rate. To assess the minimum required sample size in terms of statistical power, we used G^*^Power (39). The model of this study had six main variables. By using G^*^Power with an effect size of 0.15, alpha of 0.05, and a power of 0.95, the minimum sample size needed was only 138. Thus, we can conclude that our study with a sample size of 3,532 has a power of more than 0.95 and is large enough, indicating that the findings can be utilized with confidence.

### Research Instrument

The questionnaire consists of three sections. Demographic Information section consists of items that aim to obtain background information such as gender, age, ethnicity, economic status, education level, position, and working experience.

The 24-item Caring Behaviors Inventory (CBI-24) is considered to be the third-generation instrument for the measurement of caring (40). The current study adopts the CBI-24 by Wu et al. (41) to explore the perception of the frequency of caring behaviors as practiced by nurses. It is based upon a conceptual definition of nurse caring as an interactive and inter-subjective process that occurs during moments of shared vulnerability between nurses and patients (42). This scale consists of four components, namely, “assurance of human presence” (8 items), which deals with patients' needs and security; “knowledge and skill” (5 items), related to nurses as skillful and educated persons; “respectful deference to the other” (6 items), dealing with how nurses show interest in the patients; and “positive connectedness” (5 items), which corresponds to the need for nurses to be ready to help patients (41). For each item, respondents are requested to answer using a 6-point Likert scale (1 = never and 6 = Always). The CBI-24 demonstrated good internal consistency, Cronbach's α = 0.96 (41).

The PES-NWI is an instrument to measure the nursing practice environment in terms of ability to practice nursing skillfully and deliver high quality care (13). The 31-item PES-NWI was developed from the Nursing Work Index [NWI; (43)]. According to Lake (13), the NWI was comprised of the organizational characteristics of those hospitals that created an environment attractive to nurses but burdensome for respondents. Therefore, Lake (13) developed a PES from the NWI. The current study adopts the PES-NWI in the Malaysian context with one item removed as Malaysian nurses do not diagnose patients. PES-NWI is used to measure nurses' working environment due to it being the most commonly used and applicable instrument to measure the working environment, as well as its low respondent burden, satisfactory psychometric performance, opportunity for comparison across studies, and high discriminant ability (22, 44). The 30 items of the five NWI-based PES subscales include (i) nursing participation in hospital affairs (9 items)—the subscale reveals the participatory role and valued status of nurses in a broad hospital context, (ii) nursing foundations for quality of care (9 items)—the subscale emphasizes the nursing foundations for a high standard of patient care, (iii) nursing manager ability, leadership, and support of nurses (5 items)—the subscale focuses on the critical role of the nurse manager and ways in which they support the nurse, (iv) staffing and resource adequacy (4 items)—the subscale describes having adequate staff and support resources to provide quality patient care, and (v) nurse-physician relations (3 items)—the fifth and smallest subscale is characterized by the positive working relationships between nurses and physicians. Response for each item is given on a four-point Likert scale (1 = strongly disagree and 4 = strongly agree). The PES-NWI has established high internal reliability and consistency at the composite level (Cronbach's α = 0.82) and for each subscale (Cronbach's α ≥ 0.70).

The researchers translated the CBI-24 and PES-NWI into the Malaysian language and requested help from bilingual experts (two Malaysian nursing experts who are able to read and write in Malay and English) to translate the translated instrument (Malay version) back into the English version using back translation technique.

### Data Analysis

Partial Least Squares Structural Equation Modeling (PLS-SEM) was applied using SmartPLS 3.3.3. We employed PLS-SEM due to the inherent suitability of this approach for exploratory studies, which is the purpose of the current study (45). PLS-SEM is a comprehensive analysis approach, which can simultaneously assess the measurement model and structural model (38). The incorporation of composite second-order construct in the research framework makes PLS-SEM a suitable statistical method for the current study and to analyze the framework (38). To evaluate the conceptual model using PLS-SEM, this study evaluated the measurement model by examining the reliability and validity of reflective constructs. Meanwhile, the assessment of structural model was involved the *R*^2^, path coefficients, and the values of standardized root mean square residual (SRMR) as an approximate model fit for PLS-SEM (46).

### Validity and Reliability/Rigor

A total of 3,532 samples were used to assess the measurement and structural models. Initially, attention was focused on ensuring the reliability and validity of the reflective constructs (nursing participation in hospital affairs, nursing foundations for quality of care, nursing manager ability, leadership, and support of nurses, staffing and resource adequacy, and nurse-physician relations). This was extended to include the four reflective dimensions of caring behavior (CR): assurance of human presence, knowledge and skill, respectful deference to the other, and positive connectedness.

Next, the evaluation of reliability and convergent validity were carried out. In order to verify reliability, the threshold value of composite reliability (CR) and Cronbach's alpha (CA) should be higher than 0.7, while the minimum cutoff value for outer loading is 0.5. Also, the average variance extracted (AVE) should be higher than 0.5 (45) to confirm convergent validity. The CR and CA of all constructs in this study were above 0.70. In addition, all item loadings were above the value of 0.5, which, assuming that the CR and AVE met the required thresholds, was acceptable (45). Thirteen indicators were deleted due to their low loadings. Table 1 provides an overview of these results for all reflective constructs in stage 1, demonstrating that reliability and convergent validity had been established.

**Table 1:** Results: assessment of reflective measurement and composite models.

**Construct**	**Type**	**Items**	**Loadings/Weights**	**CR**	**AVE**	**Mean**	***SD***
Nursing participation in hospital affairs	Reflective	HA2	0.671	0.909	0.557	3.16	0.44
		HA3	0.780				
		HA4	0.823				
		HA5	0.834				
		HA6	0.791				
		HA7	0.778				
		HA8	0.659				
		HA9	0.601				
Nursing foundations for quality of care	Reflective	FQ3	0.773	0.907	0.619	3.28	0.41
		FQ4	0.796				
		FQ5	0.786				
		FQ7	0.714				
		FQ8	0.831				
		FQ9	0.816				
Nursing manager ability, leadership, and support of nurses	Reflective	NM1	0.825	0.886	0.661	3.19	0.48
		NM2	0.833				
		NM3	0.766				
		NM4	0.827				
Staffing and resource adequacy	Reflective	SR1	0.844	0.919	0.739	2.96	0.69
		SR2	0.875				
		SR3	0.865				
		SR4	0.854				
Nurse–physician relationship	Reflective	NR1	0.911	0.954	0.874	3.31	0.57
		NR2	0.948				
		NR3	0.945				
Assurance of human presence	Reflective	ASSU1	0.650	0.926	0.610		
		ASSU2	0.750				
		ASSU3	0.756				
		ASSU4	0.821				
		ASSU5	0.848				
		ASSU6	0.863				
		ASSU7	0.735				
		ASSU8	0.806				
Knowledge and skill	Reflective	KAS1	0.677	0.900	0.695		
		KAS2	0.867				
		KAS3	0.905				
		KAS4	0.867				
Respectful deference to the other	Reflective	RESPECT1	0.882	0.869	0.769		
		RESPECT2	0.872				
Positive connectedness	Reflective	CONNECT2	0.869	0.880	0.786		
		CONNECT3	0.904				
				CI_BC_0.95_	VIF		
Caring behavior	Composite	ASSU	0.413	[0.293, 0.525]	2.622	5.23	0.64
		CON	0.295	[0.179, 0.399]	2.444		
		KAS	0.218	[0.105, 0.332]	2.235		
		RES	0.234	[0.138, 0.333]	1.816		

*CR, composite reliability; AVE, average variance extracted; VIF, variance inflation factor; ASSU, assurance of human presence; NR, nurse–physician relationship; CON, positive connectedness; KAS, knowledge and skill; FQ, nursing foundations for quality of care; NM, nursing manager ability, leadership, and support of nurses; HA, nursing participation in hospital affairs; RES, respectful deference to the other; SR, staffing and resource adequacy*.

Following this, discriminant validity was examined. For this, the Fornell-Larcker criterion and heterotrait-monotrait (HTMT) approaches were employed (47). Extant research suggests that acceptable HTMT values can be lower than either 0.85 or 0.9 (48); this study adopted the 0.9 HTMT value. Table 2 shows that discriminant validity was acceptable. Further, as per the Fornell and Larcker (49) criterion, the results demonstrated that the square root of the AVE for each construct was greater than its correlation with all other constructs, again demonstrating discriminant validity (Table 3).

**Table 2:** Discriminant validity: HTMT.

**Constructs**	**ASSU**	**NR**	**CON**	**KAS**	**FQ**	**NM**	**HA**	**RES**	**SR**
ASSU									
NR	0.239								
CON	0.758	0.265							
KAS	0.802	0.221	0.661						
FQ	0.383	0.621	0.390	0.351					
NM	0.313	0.459	0.293	0.259	0.622				
HA	0.347	0.536	0.337	0.301	0.744	0.823			
RES	0.753	0.249	0.887	0.700	0.373	0.295	0.324		
SR	0.191	0.518	0.234	0.142	0.632	0.470	0.595	0.180	

*ASSU, assurance of human presence; NR, nurse–physician relationship; CON, positive connectedness; KAS, knowledge and skill; FQ, nursing foundations for quality of care; NM, nursing manager ability, leadership, and support of nurses; HA, nursing participation in hospital affairs; RES, respectful deference to the other; SR, staffing and resource adequacy*.

**Table 3:** Discriminant validity: Fornell–Larcker.

**Constructs**	**ASSU**	**NR**	**CON**	**KAS**	**FQ**	**NM**	**HA**	**RES**	**SR**
ASSU	**0.781**								
NR	0.220	**0.935**							
CON	0.621	0.219	**0.886**						
KAS	0.711	0.201	0.535	**0.834**					
FQ	0.344	0.562	0.313	0.311	**0.787**				
NM	0.274	0.402	0.230	0.223	0.531	**0.813**			
HA	0.314	0.487	0.273	0.269	0.658	0.706	**0.746**		
RES	0.601	0.201	0.635	0.548	0.293	0.226	0.260	**0.877**	
SR	0.174	0.468	0.189	0.131	0.560	0.401	0.522	0.143	**0.860**

*ASSU, assurance of human presence; NR, nurse–physician relationship; CON, positive connectedness; KAS, knowledge and skill; FQ, nursing foundations for quality of care; NM, nursing manager ability, leadership, and support of nurses; HA, nursing participation in hospital affairs; RES, respectful deference to the other; SR, staffing and resource adequacy. The bold numbers in the diagonal are the square root of AVE of each construct, and other numbers are correlations between constructs*.

Next, the measurement model of caring behavior as a second-order composite construct was assessed. To assess the measurement model of a composite construct, three criteria should be checked: multicollinearity, via variance inflation factors (VIFs), should be <5; the outer weights of associated items of the composite construct should be significant; and nomological validity should be established (50). Table 1 demonstrates that all VIF values were acceptable as they were <5 (38). Additionally, the significance of all outer weights was established via the confidence interval bias corrected approach (0.95). Further, to assess the composite construct, its nomological validity was examined (50). Following the inclusion of the composite construct, the fit indices should not be worse than prior to including them in the model (50). The SRMR for the saturated model before and after including the composite construct was 0.05, below the recommended threshold (0.08) (51), indicating an acceptable model fit and acceptable nomological validity for the composite second-order CB construct.

## Results/Findings

### Respondents' Profiles

The profiles of the respondents who participated in this survey are shown in Table 4. Out of 3,532 respondents, 3,421 (96.9%) were females. Majority of the respondents were aged 20–29 years, that is 1,395 (39.5%); whereas 50–59 years was the least number, that is 341 (9.7%). In terms of ethnicity, majority of respondents were *Kadazan* or *Dusun*, that is 1,680 (47.6%); whereas the least number was Indian, that is 20 (0.6%). With regard to level of education, majority of qualifications were at Diploma level, that is 2,096 (59.3%); and PhD was the smallest number, that is 1 (0.1%). For economic status, majority of respondents described their economic status as medium, that is 2,486 (70.4%); and luxurious was the smallest number, that is 9 (0.3%). Regarding positions in nursing, majority of respondents were Staff Nurse U29, that is 1,795 (50.8%); and the least number was the Head Nurse (Nursing Sister) U41, that is 3 (0.1%). With regard to working experience, majority of the respondents had <5 years of working experience, that is 1,229 (34.8%); whereas the least number was more than 35 years, that is 33 (0.9%).

**Table 4:** Demographic profile of respondents.

		**Frequency**	**%**
Gender	Female	3,421	96.9
	Male	111	3.1
Age	20–29 years old	1,395	39.5
	30–39 years old	1,214	34.4
	40–49 years old	582	16.5
	50–59 years old	341	9.7
Ethnicity	Kadazan/Dusun	1,680	47.6
	Bajau	404	11.4
	Malay Brunei	233	6.6
	Others Bumiputera	739	20.9
	Malay	197	5.6
	Chinese	73	2.1
	Indian	20	0.6
	Others non-Bumiputera	186	5.3
Level of education	PhD	1	0.1
	Master's degree	7	0.2
	Bachelor's degree	135	3.8
	Diploma	2,096	59.3
	Certificate	1,293	36.6
Economic status	Low	205	5.8
	Below average	621	17.6
	Medium	2,486	70.4
	Above average	211	6.0
	Luxurious	9	0.3
Positions	Nurse Supervisor (Matron) U42	9	0.3
	Nurse Supervisor (Matron) U41	5	0.1
	Head Nurse (Nursing Sister) U41	3	0.1
	Clinical Nurse Specialist U41	5	0.1
	Nurse Supervisor (Matron) U36	30	0.8
	Head Nurse (Nursing Sister) U32	406	11.5
	Staff Nurse U29	1,795	50.8
	Community Nurse U26	22	0.6
	Community Nurse U24	148	4.2
	Community Nurse U19	1,072	30.4
	Assistant Nurse U14	22	0.6
	Assistant Nurse U11	15	0.4
Working experience	>35 years	33	0.9
	30–35 years	124	3.5
	25–29 years	253	7.2
	20–24 years	187	5.3
	15–19 years	313	8.9
	10–14 years	578	16.4
	5–9 years	815	23.1
	<5 years	1,229	34.8

### Structural Model

According to Table 1, the mean scores and standard deviations (*SD*) for our study variables were 5.23 for caring behavior (*SD* = 0.64); 3.16 for nursing participation in hospital affairs (*SD* = 0.44); 3.28 for nursing foundations for quality of care (*SD* = 0.41); 3.19 for nursing manager ability, leadership, and support of nurses (*SD* = 0.48); 2.96 for staffing and resource adequacy (*SD* = 0.69); and 3.31 for nurse-physician relations (*SD* = 0.57).

Before assessing the structural model, the collinearity between research variables was evaluated to ensure that the structural model did not include any lateral collinearity issue (38). Table 5 shows that all inner VIF values were below 5 (38), indicating that collinearity among the predictor constructs was not a concern in the structural model. Next, we assessed the structural model by computing the path coefficient, *t*-values, and *R*^2^ using a 5,000 sampling bootstrapping technique (38). Finally, the predictive relevance (*Q*^2^) was examined.

**Table 5:** Results of hypothesis testing.

**Hypothesis**	**Direct effect**	**Path coefficient**	***t*-value**	**95% CI**	**Supported**	**VIF**
Hypothesis 1	HA → CB	0.135	5.140	[0.083, 0.187]	Yes	2.693
Hypothesis 2	FQ → CB	0.274	12.885	[0.232, 0.314]	Yes	2.218
Hypothesis 3	NM → CB	0.063	2.726	[0.017, 0.108]	Yes	2.023
Hypothesis 4	SR → CB	−0.085	4.574	[−0.122, −0.049]	No	1.607
Hypothesis 5	NR → CB	0.040	2.039	[0.004, 0.079]	Yes	1.571

*NR, nurse–physician relationship; FQ, nursing foundations for quality of care; NM, nursing manager ability, leadership, and support of nurses; HA, nursing participation in hospital affairs; SR, staffing and resource adequacy; CB, caring behavior*.

**Figure 2:**
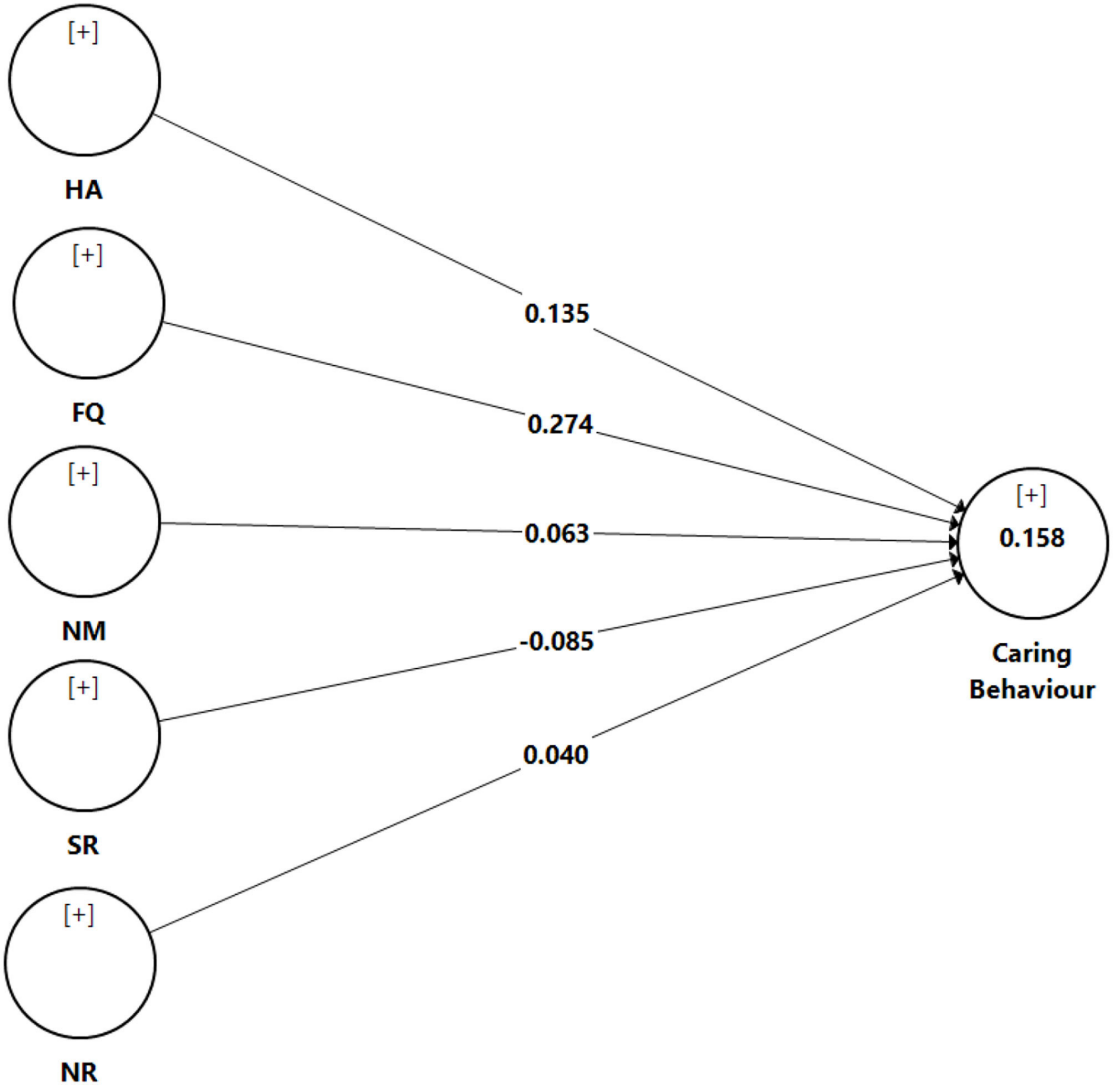
Results: assessment of structural model. NR, nurse–physician relationship; FQ, nursing foundations for quality of care; NM, nursing manager ability, leadership, and support of nurses; HA, nursing participation in hospital affairs; SR, staffing and resource adequacy.

Based on Table 5 and Figure 2, nurse participation in hospital affairs (β = 0.135, *t* = 5.140, *p* <0.05), nursing foundations for quality of care (β = 0.274, *t* = 12.885, *p* <0.05), nurse manager ability, leadership (β = 0.063, *t* = 2.726, *p* <0.05), and support of nurses, and nurse-physician relations (β = 0.040, *t* = 2.039, *p* <0.05) showed a positive significant effect on caring behavior. Meanwhile, it was found that staffing and resource adequacy (β = −0.085, *t* = 4.574, *p* <0.05) had a negative significant effect on caring behavior. In short, all direct hypotheses were supported except Hypothesis 4. With regard to the *R*^2^-value, the results show an *R*^2^-value of 0.158 for caring behavior, suggesting that 15.8% of the variance for caring behavior can be described by nursing participation in hospital affairs, nursing foundations for quality of care, nursing manager ability, leadership, and support of nurses, staffing and resource adequacy, and nurse-physician relations. An *R*^2^-value of 0.158 is considered moderate for behavioral studies (38).

In addition, the blindfolding technique was implemented to assess the predictive relevance, which is only used for reflective endogenous constructs (38). The predictive relevance of cross-validated redundancy values (*Q*^2^) for the endogenous variables is 0.114, which is higher than zero. Therefore, the model had predictive relevance for the outcome variables (45).

## Discussion

The aim of this study was mainly to examine the impact of nurses' working environments on their caring behavior in public hospitals and public health services throughout Sabah. Our findings show that nurses' perception on the work environment is partially linked with nurses' caring behavior, supporting hypotheses H1, H2, H3, and H5. These results correspond with previous studies which have consistently shown that positive perceptions of work environment are linked with nurses' higher quality of care (25, 29, 30, 34–36). These results also align with the main assumption of the NWLM domains as important in supporting nurses' work environment to enhance the quality of nurses' caring behavior. However, staffing and resource adequacy has a negative effect on caring behavior, thus rejecting Hypotheses H4. This study has reinforced the suggestion of Finfgeld-Connett (12) that the working environment must be conducive to nurses, with sufficient resources and time to carry out proper nursing care.

The results of this study suggest that nurses who report favorable nursing participation in hospital affairs, nursing foundations for quality of care, nursing manager ability, leadership, and support of nurses, and nurse-physician relations are more likely to report better caring behavior (25). As documented in the literature, nurses, direct-care, managers, and executive leadership are urged to participate in collective decision-making throughout all levels of the organization to establish an empowered working environment. A desirable work environment with sufficient support, which is consistent with the professional structure, empowers nurses to practice to the full scope of their knowledge, competencies and skills in patient care (52). In such an environment, nurses are more satisfied and provide higher quality of care (53). Previous empirical evidence has shown that nurses who reported favorable nursing foundations for quality of care were less likely to report burnout and leave their current position, suggesting that better practice environments can help to achieve optimal nursing care (35). Healthy work environments reduce burnout and promote caring relationships, which are in turn, essential in order to accomplish patient-centered care, highly functioning healthcare teams, engaged nurses, and satisfied patients and families. Pertaining to this study, in order for nurses to have quality caring behavior, they need higher nursing foundations for a high standard of patient care (a pervasive nursing philosophy), a nursing (rather than a medical) model of care, clinical competence, and a formal quality assurance program. Also pertinent are the cultivation of new staff and continuing education for all staff, and continuity of nursing care and the use of nursing diagnoses and nursing care plans.

This study is also consistent with that by Oluma and Abadiga (17). Their study supports the notion that nurses' caring behaviors are significantly associated with nursing leader management, staffing and support. This shows that nurses who perceive the work environment as empowering tend to provide high levels of caring behavior. In addition, Zaghini et al. (54) confirmed that when nurses were satisfied with leadership, they felt less burned out or tensed in interpersonal relationships, were less involved in bad behavior, and, in turn, patients were more satisfied with the quality of care provided. In addition, Smith et al. (55) also confirmed that the quality of the nurse manager is a major factor in the work environment of nurses with regard to bad manners. The horizontal hostility among nurses is normal in the workplace and was reported to have adverse impact on quality of care (56). In this occurrence, we're talking about nurses who turn on one another when they ought to have each other's backs. Horizontal hostility generally takes in the form of psychological harassment which include gossip and backstabbing. When hostility becomes an issue in the work environment, the manager, supervisor, or an administrator need to become more involved and being noticeable in offering support to nurses who are being hostility victimized (57).

This study also corroborates previous studies on the effect of nurse–physician relations on unit level quality of nursing care and personal accomplishments (25, 56, 58–60). The more collegial the nurse-physician relationship, the lower the number of patient complaints reported (34). Thus, to enhance quality of care, there should be effective communication, collaboration and decision-making, along with recognition of everyone's contributions. As asserted by Cassidy (26), hospitals and healthcare systems are relationship-based human systems, and it is therefore suggested that nurses, other members of the healthcare team, patients, and families work in synchrony to achieve healthcare aims. On the other hand, Kaifi et al. (61) examined four domains (shared education and teamwork, caring vs. curing, nurses' autonomy, and physicians' dominance) which were identified as the factors influencing inter-professional collaboration. They found that nurses had significantly better opinions about inter-professional collaboration than doctors. Nurses also outperformed doctors in all four domains (education and collaboration, caring vs. curing, nurse's autonomy, and physician's authority). The results showed that nurses valued inter-professional collaboration more than doctors. The researchers also suggested that inter-professional collaboration through educational methods could help bridge the gap in different mindsets.

Evidence related to the skill mix of the nursing team pointed to either no benefit or a negative effect, as observed from the higher levels of support workers (62). Nevertheless, the results of this study indicate that staffing and resource adequacy negatively influences nurses' caring behavior. In fact, the current study validates the Moisoglou et al. (63) study which found that higher staffing and resource adequacy was linked with frequent/very frequent patients' falls, medication errors, deep venous thrombosis and ulcers as frequent/very frequent safety indicators, despite scoring higher staffing, and resource adequacy. Although nurse staffing has been reported to influence the quality of nursing care and patient outcomes (32), the causal link between nurse staffing levels and outcomes remains disputed (64). Certainly, for most patient outcomes the causal association can only be partial and indirect. In their umbrella review on the relationship between nurse staffing levels and nursing-sensitive patient outcomes, Blume et al. (65) concluded that it was difficult to draw a reliable statistical conclusion due to the large number of aspects that could influence the impact of hospital staffing on patient outcomes. Twigg et al. (66) in their systematic review further asserted that the previous literatures regarding staffing methodologies cannot highlight to any methodology as being prevalent in improving patient and nurse outcomes. Rather, their review found that the improvements in nurse staffing levels has the related advantages of improving nurse and patient outcomes. Nurse staffing is often measured by nurse-to-patient ratio, nurse hours per patient day, and perceived adequate nurse staffing (64), but the most appropriate nurse staffing measures are found to be nursing hours per patient day and nurse-to-patient ratio using a Delphi survey (67). However, these staffing methodologies are yet to decide how many hours provided by a nurse is needed in order to provide quality care (66). Consequently, there is a need of relative investigations to be led to characterize the staffing parameters required in order to have an impact on quality care.

### Limitations

This study is novel in its investigation of the role the working environment plays in nurses' caring behavior. However, as with any piece of research, limitations exist. First, all questionnaires were distributed through the Matron, Head Nurse (Nursing Sister), Staff Nurse or Community Nurse assigned to the public hospital and public health services for data collection. These were then redistributed to the respondents, thus passing through various levels. As a result, confidentiality during the process of data collection was compromised to some degree, as it was beyond the control of the researchers, seeing that they did not have the opportunity to administer face-to-face data collection. Therefore, future researchers should explore the work environment and caring behavior separately, as the Head Nurse, Staff Nurses, Community Nurses, and Public Health Nurses carry out different tasks which are set according to their positions. Second, the respondents were nurses from both public hospitals and public health services which have different categories of staff who are given different task responsibilities based on their qualifications. Hence, differences in nurses' caring behaviors may be found if a comparative analysis was performed. In order to generalize the results, future studies should investigate according to the discipline nurses are assigned to, such as nurses working in hospitals and nurses working in healthcare services, as different settings may have different work environments.

### Implications

The present research has provided convergent evidence on the role of the working environment in influencing the behavior of nurses working in hospitals and health clinics in Sabah, Malaysia.

To support nurses in term of participation in hospital affairs, nurse managers should consider the appointment of senior nursing administrators who are highly visible and accessible to staff, administrators who listen and respond to employee concerns, nursing administrators who consult with staff on daily problems and procedures and senior nursing administrators equal in power and authority to other top level hospital executives. In addition, policymaker should consider to provide nurses the opportunities for career development/clinical ladder prospects, advancement, serving on hospital and nursing committees, participating in policy decisions, and involvement in the internal governance of the hospital.

In terms of nursing foundations for quality of care, policymakers should ensure active staff development or continuing education programs for nurses. There should also be more nurses with a Bachelor of Science in Nursing and degree-holders staff nurses who are clinically more competent so as to provide a balance to a workforce of majority diploma-level nurses. Alternatively, there should at least be clinically competent nurses functioning as a reference source or as nursing care team leaders. Given the focus on quality outcomes and the need for safe patient care in the contemporary healthcare environment, registered nurses need professional development training that enhances their ability to provide safe and high-quality care. Therefore, nursing managers should encourage and give opportunities to their subordinates to engage in active staff development or continuing education programs, so that nurses acquire updated knowledge and skills.

In terms of nursing manager ability, leadership, and support of nurses, policymakers should put in place managers or immediate supervisors with good management and leadership skills who support the nursing staff in decision-making (even if there is conflict with doctors), use mistakes as learning opportunities, not criticism, are supportive of nurses and give praise and recognition for a job well done. To improve the nurse-physician relationship, nurse managers should create environments that build good doctors and nurses working relationships, as well as teamwork and collaboration between nurses and doctors.

## Conclusion

This study is greatly significant in that it offers insights into the influence of work environment on nurses' caring behavior in the context of Sabah, Malaysian. In light of this, our study has revealed that in the nursing working environment, factors like participation in hospital affairs; foundations for quality of care; manager ability, leadership, and support of nurses; and nurse-physician relations are imperative. The nursing foundations for quality of care in particular is found to have the greatest impact. Nevertheless, as most public hospitals and public health services in Malaysia are still struggling with issues related to nursing working environments (3), the challenges they face in deliver top notch services to the people have proven to be a stumbling block. Thus, it is important for Malaysian public hospitals and public health services to take appropriate measures and make effective decisions to overcome these challenges in order to be successful in providing good environment for nurses to practice at workplace and ultimately increase quality of care. Our results also resonate with the assertion of the NWLM which painted a holistic composition with regard to the nursing work environment. Our study, notably, has extended the scope of this model by examining factors such as cohesive teams, collegial relationships, professional development, and supportive leadership and identifying them as the forces that drive nurses' caring behavior in the context of Nurses in Sabah, Malaysia. More studies are warranted to be conducted beyond the Sabahan nurses' perspectives, especially to determine the relationships between nurse staffing, resource adequacy and caring behavior.

## Data Availability Statement

The raw data supporting the conclusions of this article will be made available by the authors, without undue reservation.

## Ethics Statement

The studies involving human participants were reviewed and approved by Medical Research and Ethics Committee (MREC), and National Medical Research Register (NMRR) (ref no: NMRR-14-1226-21410) of Malaysia, Ministry of Health (KKM) and Sabah State Health Director [ref no: JKN(SB)PJNS/32]. The patients/participants provided their written informed consent to participate in this study.

## Author Contributions

NA, BC, WW, and ND made substantial contributions to conception and design, or acquisition of data, or analysis and interpretation of data. NA and WW involved in drafting the manuscript or revising it critically for important intellectual content, and agreed to be accountable for all aspects of the work in ensuring that questions related to the accuracy or integrity of any part of the work are appropriately investigated and resolved. BC and NA given final approval of the version to be published. All authors contributed to the article and approved the submitted version.

## Funding

The authors gratefully acknowledge the help and support of the hospital directors, matrons, and all staff nurses of the participating hospitals. The authors also wish to extend their gratitude to Universiti Malaysia Sabah for funding this research (Grant: SLB0106-SS-2015).

## Conflict of Interest

The authors declare that the research was conducted in the absence of any commercial or financial relationships that could be construed as a potential conflict of interest.

## Publisher's Note

All claims expressed in this article are solely those of the authors and do not necessarily represent those of their affiliated organizations, or those of the publisher, the editors and the reviewers. Any product that may be evaluated in this article, or claim that may be made by its manufacturer, is not guaranteed or endorsed by the publisher.
